# Customized Technological Designs to Improve the Traditional Use of *Rosa canina* Fruits in Foods and Ingredients

**DOI:** 10.3390/plants12040754

**Published:** 2023-02-08

**Authors:** Adina Andreea Teodorescu, Ștefania Adelina Milea, Bogdan Păcularu-Burada, Oana Viorela Nistor, Doina Georgeta Andronoiu, Gabriela Râpeanu, Nicoleta Stănciuc

**Affiliations:** Faculty of Food Science and Engineering, Dunărea de Jos University of Galati, Domnească Street 111, 800201 Galati, Romania

**Keywords:** rosehip fruits, plant-based food, antioxidant activity, carotenoids, jellified products, juices, nutraceuticals

## Abstract

The rosehip fruits from Romanian spontaneous flora were valorized in three different customized variants, including jellified products, juices, and a nutraceutical. Prior to the technological design, the rosehip samples were characterized for individual polyphenolic content. The samples (non)-enzymatically treated showed epicatechin as the major compound, whereas the enzymatic treatment enhanced the extraction of quercetin (40.23 ± 0.48 mg/100 g DW extract), gallic acid (9.74 ± 0.24 mg/100 g DW extract), and kaempferol. Different ratios and types of sugars were added to obtain jellified products, leading to a significantly different bioactive profile. The sugar-free and steviol samples showed the highest content in carotenoids (89.36 ± 0.06 mg/g dry weight (DW) and 39.22 ± 1.16 mg/g DW, respectively), leading to the highest antioxidant activity (8.19 ± 0.80 mMol Trolox/g DW and 20.16 ± 0.31 mMol Trolox/g DW, respectively). The gelling capacity increased with the increase in sugar content. The enzymatically treated rosehip fruit pulp was mixed in various ratios with apple juice, followed by pasteurization. The polyphenolic content was approximately two times higher in the blended juices (20.75 ± 1.40 mg gallic acid equivalents (GAE)/100 mL). The overall impression highlighted the preferences of panelists for sugar-free products, whereas adding apple juice significantly influenced their preferences. The fresh pulp was enhanced with pectin, followed by inoculation with *Lactobacillus acidophilus*, and freeze-dried showed satisfactory cell viability (approximately 7 log CFU/g DW), whereas an antidiabetic, anti-tyrosinase and anti-obesity potential of the powder was suggested. Our results provide enough evidence for customized processing of rosehip fruits in foods and nutraceuticals as a response to consumers’ choices, highlighting the bioactive compounds and nutrient contents, whereas selected in vitro health-related evidence was suggested.

## 1. Introduction

Significant efforts, both from scientific and technological perspectives, are given to develop new alternative, customized technological processes for food able to fulfill the sensory and functional requirements of the consumers while satisfying the producers’ search for foods with a value-added, from the technological and economic perspective [1]. The development of new plant-based foods is needed, considering the systematic supply of bioactive compounds, such as essential fatty acids, tannins, minerals, vitamins, carotenoids, phenolic compounds, fiber, etc., in order to provide a wide spectrum of nutritional and biological benefits for a healthy diet [2]. Rosehip fruits represent an alternative to plant-based food production since the fruit`s health-associated properties are well documented [3,4,5].

Rosehip (*Rosa canina* L.) are reddish-color fruit that appears in an aggregate form on shrubs, belonging to the *Rosa* genus of the *Rosaceae* family, and comprises nearly 200 species with complex taxonomy [6]. Rosehip shrubs are widely distributed, mainly in temperate to tropical habitats of Europe, Asia, the Middle East, and North America [7]. Rosehip fruits are traditionally used against many diseases, such as immunosuppressive, antioxidant, anti-inflammatory, anti-arthritic, analgesic, anti-diabetic, cardioprotective, antinociceptive, antimicrobial, gastroprotective, and skin ameliorative effects [8]. The processing potential of rosehip fruits is high, considering the wide spectrum and richness in bioactive compounds, such as phenolics, carotenoids, essential oils, different vitamins, such as C, B1, B2, K, A, minerals (calcium and potassium), pectin [9]. Due to the slightly tart taste of its fresh fruit, short harvesting season, and sensitivity to storage, fresh rosehip fruits are processed as tea, jam, nectar, and marmalade [10]. In Central European countries’ rosehips are traditionally used as herbal teas or processed foods, such as jellies and marmalades, yogurts, jams, soups, food supplements, wine, and nectars [11]. Rosehip fruits contain high amounts of ascorbic acid content, ranging from 3 g/kg to 40 g/kg [12], thus showing a significantly higher content compared to other commonly consumed fruits or vegetables [13].

Considering the richness and the diverse spectrum of bioactive compounds, rosehips show great potential for the development of functional food with nutritional, functional, and economic value. Therefore, this study proposed three customized technological designs for the processing of rosehip fruits into marmalade, juices, and a probiotic-based nutraceutical. Hereto, the following technological approaches were used: (i) processing of fresh rosehip fruits into jellified foods, with different ratios and types of sugars addition, in order to customize the technological process for designing foods for breakfast, cookies, cakes, and chocolates as confectionary products; (ii) enzymatically processing of fresh rosehip fruits to increase the yield of bioactive compounds in juice, in several variants, with and without combination with other juices; (iii) inoculation of rosehip fruits pulp with selected lactic bacteria followed by freeze-drying, to obtain a nutraceutical with multifunctional properties. 

The rosehip jellified products were tested for carotenoid content and antioxidant activity, texture, color, and acceptability test, whereas the juices were analyzed for carotenoids and polyphenols content, antioxidant activity, and acceptability test. The fresh rosehip fruit pulp was also inoculated with *Lactobacillus acidophilus* and freeze-dried to obtain a powder for nutraceutical uses. The powder was tested for phytochemicals content (carotenoids and polyphenolic contents), antioxidant activity, structure and morphology, and the ability to inhibit selected metabolic syndrome-associated enzymes, such as α-glucosidase and lipase. The potential of the powder to inhibit the enzymatic activity of tyrosinase was also tested.

## 2. Results and Discussion

### 2.1. The Polyphenolic Content of the Extracts

**Figure 1:**
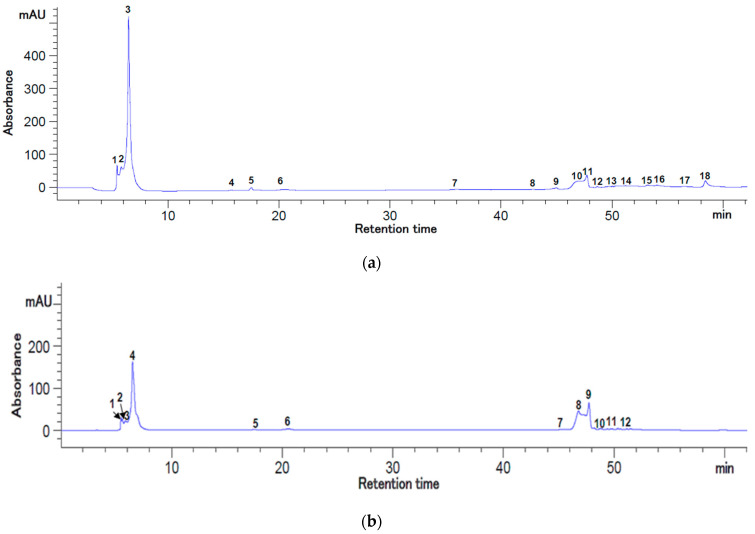
HPLC chromatograms for the flavonoids and polyphenols from rosehip’s extract (1 M) at 280 nm (**a**) and 320 nm (**b**). Peaks’ identification: (**a**) 1—gallic acid; 2—chlorogenic acid; 3—epicatechin; 10—quercetin; 14—kaempferol, 4–9, 11–13, 15–18—unidentified peaks; (**b**) 2—gallic acid; 3—chlorogenic acid; 4—epicatechin; 8—quercetin 3-*β*-D-glucoside; 9—quercetin; 12—kaempferol; 1, 5–7, 10, 11—unidentified peaks.

**Figure 2:**
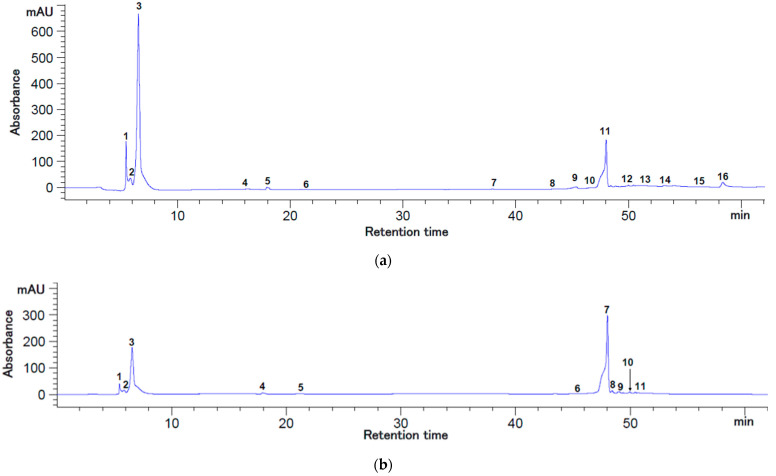
HPLC chromatograms for the flavonoids and polyphenols from rosehip’s extract (2 M) at 280 nm (**a**) and 320 nm (**b**). Peaks’ identification: (**a**) 1—gallic acid; 2—chlorogenic acid; 3—epicatechin; 10—quercetin 3-β-D-glucoside; 11—quercetin; 13—kaempferol, 4–9, 12, 14–16—unidentified peaks; (**b**) 1—gallic acid; 2—chlorogenic acid; 3—epicatechin; 7—quercetin; 11—kaempferol; 4–6, 8–10—unidentified peaks.

The HPLC analysis of the bioactive compounds from the rosehip berries’ extracts presented in Figure 1 and Figure 2 suggests that the enzymatic treatment of the rose-hip pulp (extract coded RE) induced significant differences (*p* < 0.05) among some individual bioactive compounds’ concentrations.

The RE extract was characterized by an increased amount of quercetin (40.23 ± 0.48 mg/100 g DW extract), gallic acid (9.74 ± 0.24 mg/100 g DW extract), and kaempferol (5.06 ± 0.05 mg/100 g DW extract). However, epicatechin was the major compound identified in both extracts, with variations between 2077.22–2145.04 mg/100 g DW extract (*p* > 0.05), followed by chlorogenic acid with a concentration of 23.12 ± 1.67 mg/100 g DW in RE extract, respectively 39.65 ± 9.12 mg/100 g DW in R extract (Table 1).

The bioactive compounds separated from rosehip berries and their functionality are strongly linked to enzymatical pretreatment. Gallic acid amounts between 2.77–84.89 mg/100 g DW, respectively chlorogenic acid (3.21–22.10 mg/100 g DW), and quercetin (1.37–2.28 mg/100 g DW) were identified in rosehip fruits with different ripening levels from different cultivars [14]. Gallic and chlorogenic acids, kaempferol, or quercetin glucosides were also identified in rosehip fruits’ pulp or seeds [9]. 

### 2.2. The Carotenoid Profile of the Jellified Products

It has been suggested that jellified products are still favorite by consumers [15], generally obtained by gelatinization in the presence of pectin, agar or gelatin, sugar flavorings, acids, and dyes. However, this category of food contains a high concentration of sugars, displaying a low nutritional value, whereas excessive consumption can cause dental cavities, obesity, and hyper-glycaemia [16]. Therefore, different approaches to reduce the proportion or to completely substitute the sugar may bring significant health-associated benefits [16]. In this study, rosehip pulp was processed in four variants of jellies, with and without sugar and sugar substitutes. 

The jellified products were analyzed for carotenoid content and antioxidant activity (Table 2). For the sake of comparison, the phytochemical analyses also included the blank sample represented by the rosehip pulp (P) and all four variants of processed samples. The unprocessed pulp (P) showed a total carotenoid content of 185.17 ± 1.68 mg/g DW, with a β-carotene and lycopene content of 148.98 ± 1.06 mg/g DW and 81.16 ± 2.42 mg/g DW, respectively. The carotenoid content in processed samples was significantly influenced by the sugar and pectin addition. For example, the jellified product without the addition of sugar showed the highest carotenoids content (Table 2), suggesting that during the heating of the sample coded as V, the concentration of the sample due to the heat treatment led to a significant decrease in the carotenoids content, when compared with the unprocessed sample. The carotenoids content in samples with the sugar and pectin addition was affected by the specific network formed during heat treatment, with the highest value the for sample with steviol addition (V3), whereas in the samples with sugar addition (V1 and V2), the carotenoid content varied in a linear-depended manner with the added sugar concentration. The results are highly correlated with the texture profile of jellified products.

During thermal treatment and jelling of the samples, the complex network formed between sugar, pectin, and other macromolecular polysaccharides significantly had a significant effect on the carotenoid content in terms of total carotenoid, β-carotene, and lycopene, impacting the apparent viscosity of the samples. The untreated pulp showed the highest carotenoid content, followed by the variant without sugar addition (Table 2). The sugar addition led to a decrease in carotenoid content in a concentration-dependent manner. Based on the considerable thermal (up to 80 °C) and pH (2–10) stability of stevia, a potential protective effect of the steviol glycosides on carotenoids could also be suggested [17]. The phytochemical profiles highly influenced the antioxidant activity values, with the highest antiradical capacity for P of 36.44 ± 2.45 mMol Trolox/g DW, whereas the jellified samples showed values of 20.16 ± 0.31 mMol Trolox/g DW in V3, 8.19 ± 0.80 mMol Trolox/g DW in V and no significantly different values for V1 and V2 (Table 2). The carotenoid content in the jellified rosehip products was significantly lower than those reported by Yildiz and Alpaslan [11] (88.05 mg/kg), whereas the antioxidant capacity was 41.25 mM Trolox/g for the samples obtained using the classical method.

### 2.3. The Texture Analysis of the Jellified Products

The textural parameters determined by TPA were described by Bourne [18] as firmness (N), adhesiveness (mJ), and cohesiveness (mm). The values of the parameters are given in Table 3. The highest firmness values were registered for the samples with the added sugar, 3.62 ± 0.08 N and 2.85 ± 0.03 N for V1 and V2, respectively. This could be explained by the complex gel matrix achieved as a result of the interaction between sugar, pectin, and acids [19]. Steviol shows low gelling capacity, with results from the firmness value (1.60 ± 0.02 N), almost the same as the one obtained for the sample without sugar (1.14 ± 0.02 N). 

At the same time, the samples with added sugar show the highest adhesiveness, cohesiveness, and springiness values, indicating greater spreading resistance. 

### 2.4. Color Parameters of the Jellified Products

Color is an important quality parameter because it reflects the changes occurring during chemical reactions, degradation, or thermal processing [20]. The color of all the jellified samples was determined to evaluate the effect of sugar addition and thermal treatment. The results are presented in Table 4. 

As expected, the control sample, represented by raw rosehip fruits, registered the highest values for lightness (L* of 32.10 ± 0.5) and redness (a* of 6.39 ± 0.1). Similar values (L* of 29.61 ± 0.7 and a* of 7.01 ± 0.1) were determined for V3, which is mainly related to the preservation of lycopene content of rosehip during processing sustained by the steviol addition.

Moreover, the values for L* (22.39 ± 1.00 and 29.61 ± 0.70), a* (9.89 ± 0.20 and 7.01 ± 0.3), and b* (28.44 ± 0.2 and 17.86 ± 1.00) parameters for V and V3 samples are in accordance with Yildiz and lpaslan [11] for the rosehip marmalades produced by using vacuum evaporator, which is a commercial preferably method for these types of products. According to Drożdż et al. [21], the increase in a* value in all the samples, except V1, is due to the exposure of anthocyanin and natural carotenoid compounds of rosehip after the thermal treatment. Comparable values for all the color parameters were registered for samples V1 and V2 with sugar addition. A decrease of about 11 and 16% in the lightness of V1 and V2 indicates the changes induced by the temperature and cooking time, with an impact on the sugar caramelization. In conclusion, the addition of stevia sugar is the best technological option sustained by the positive impact on the color of rosehip jellified products.

### 2.5. Proximate Chemical Composition of the Jellified Products

The proximate analysis and caloric values of the jellified samples are shown in Table 5. As can be observed, different nutrient levels in the samples were found based on the customized applied approach (Table 5). Not surprisingly, there was an increase in the moisture and ash in the jellified products, which was correlated with the percentage of sugar added (*p* < 0.05). In the samples with ratios of 1:1 and 2:1 addition of sugar, a significant decrease in the moisture content was found, which corresponds to the water binding in the specific networks formed between sugar and pectin during heat treatment. The sodium levels were similar for samples V and V3, whereas no significant levels were found for samples V1 and V2, whereas the V samples showed a higher level of fats. In samples without sugar addition, the constant level of salt may be correlated with the textural properties, whereas the increase in firmness led to a concentration of the samples with a slight increase in sodium content.

Protein levels remained unaffected, whereas carbohydrate levels significantly differed in a linear-depended manner with the concentration and type of the added sugar (Table 5). Consecutively, there was a significant decrease in caloric values of the samples without sugar, pectin, and steviol addition, respectively.

Yildiz and Alpaslan [11] suggested a significantly higher protein content in marmalades obtained from rosehip fruits by using a different concentration method, with the highest content of 3.3% for the vacuum evaporator method and the lowest for the commercial samples. These authors also suggested a significantly lower content in ash (up to 0.25%), while the total sugar content was higher for the commercial, at 54.27%.

### 2.6. The Carotenoids and Polyphenolic Profile of the Rosehip Juices

Enzymatic treatment of the rosehip pulp was applied in our study in order to increase the juice yield, stabilize the specific color, and improve the pulp liquefaction while decreasing turbidity and viscosity. In rosehip fruits, pectin is involved in crosslinking cellulose and hemicellulose fibers. Therefore, the pectinases and hemicellulases blend used in our study allowed the access of enzymes to their corresponding substrates [22], thus allowing liquefying of the pulp and preserving the specific color of the fruit. The pulp and juices were characterized for carotenoid and antioxidant activity (Table 6). 

The resulting pulp was further processed in juices, with and without apple juice addition. The carotenoid content varied significantly, with a higher value in the J sample. The polyphenols content varied in a linear-dependent manner with the concentration of apple juice addition (Table 6). Due to the higher polyphenolic content in J2, the antioxidant activity was significantly higher (20.75 ± 1.40 mMol Trolox/100 mL). Pashazadeh et al. [23] studied the antioxidant capacity, phytochemical compounds, and volatile compounds related to the aromatic property of vinegar produced from black rosehip (*Rosa pimpinellifolia* L.) juice. These authors reported a higher total polyphenolic content of the juice before fermentation of 10.59 mg GAE/mL and a related antioxidant activity of 78.83 mMol Trolox/mL.

### 2.7. The Acceptance Test for Jellified Products and Juices

One of the most important aspects related to the new food formulations consists of the consumer appeal by using different rating scales for the degree of liking or disliking, reflecting, therefore, the preference degree of consumers regarding the food product. The acceptance analysis of the jellified products was performed following the different sensorial characteristics such as appearance, color, sweet taste, flavor, bitter taste, hardness, fragility, gumminess, mouthfeel, creaminess, and general impression (Table 7). 

On the other hand, the sensorial analysis for the juices (Table 8) involved the following attributes appearance, color, sweet taste, bitter taste, flavor, and general impression. 

As expected, the sweet taste and mouthfeel were best scored on the sample with the highest sugar content. Color retention is obviously best associated with the sugar-free sample. Regarding the overall impression, samples with a lower sugar content were the most appreciated, which represents an advantage both on health by preventing hyperglycemia, and also on the costs necessary to make the jellified product. 

The rosehip pulp juice without the addition of apple juice (variant J) was better scored regarding appearance and color. The addition of apple juice improved the sweet taste and flavor due to the apple’s natural composition. Overall impression obtained a high score for all the samples, increasing with the increase in the apple juice content. The slightly increased bitter taste of sample J could be attributed to the astringency of rosehips. These results are highlight rosehips as a potential source of food ingredient that deserves to be integrated into food products or drinks in everyday diet.

### 2.8. The Phytochemical Characterization and Cell Viability of the Inoculated Powder

The global phytochemical profile of the powder exhibited a TFC of 14.78 ± 1.52 mg CE/g DW and TPC of 52.10 ± 0.61 mg GAE/g DW, whereas the content of carotenoids was 15.38 ± 0.02 mg total carotenoids/g DW, with a β-carotene content of 11.78 ± 0.01 mg/g DW and lycopene of 9.84 ± 0.01 mg/g DW, respectively. This high concentration of biologically active compounds yielded a DPPH radical scavenging activity of 6.59 ± 0.20 mM Trolox/g DW and a radical inhibition of 75.62 ± 2.40%. Cell viability in powder was 6.81 log CFU/g DW; nevertheless, the survival rate of *Lb. acidophilus* after freeze-dying was calculated to be 82.73 ± 0.60%. In other studies, Turgut et al. [24] added rosehip marmalade into a yogurt fermented with *Lb. acidophilus* and showed a decrease of 2.2 log CFU/g during storage. Milea et al. [25] incorporated *Lb. casei* in a microencapsulated extract of flavonoids from yellow onion skins and obtained an efficiency of 72.49 ± 0.11% regarding the cells` viability.

### 2.9. The Inhibitory Activity of the Powder 

The inhibitory ability of the powder on the two main digestive enzymes (pancreatic lipase and α-glucosidase) and tyrosinase is summarized in Table 9. The powder showed the strongest inhibitory activity towards tyrosinase, followed by pancreatic lipase and α-glucosidase (Table 9). Therefore, the IC50 value describing the inhibition activity against α-glucosidase was 2.92 ± 0.83 mg/mL, approximately 27% lower than that of acarbose (3.97 ± 0.62 mg/mL). The pancreatic lipase inhibitory activity of the inoculated powder showed a value of 1.21 ± 0.08 mg/mL, comparable with the corresponding value for Orlistat, 1.23 ± 0.09 mg/mL.

In the case of tyrosinase, the corresponding IC50 value of powder was 0.23 ± 0.05 mg/mL, significantly higher than the corresponding value for the kojic acid (1.12 ± 0.14 mg/mL), thus illustrating the higher effectiveness on inhibiting the tyrosinase. 

### 2.10. Microscopic Structure of the Powder

**Figure 3:**
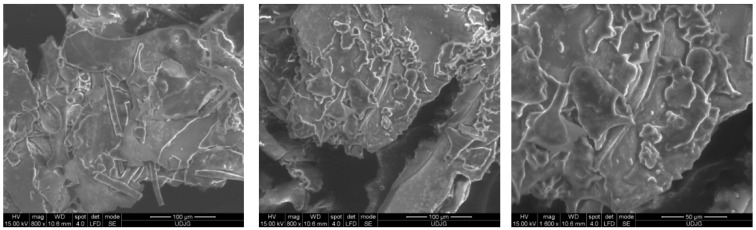
Scanning electron microscopy micrographs of freeze-dried rosehip pulp inoculated with *Lactobacillus acidophilus*.

The microstructure of the inoculated powder defines important physical features and the potential controlled release of the bacterial load and bioactive compounds. Scanning electron microscopy (SEM) was used to visualize the architecture of the network formed in the structurally complex powder matrices (Figure 3).

From Figure 3, it can be observed that the continuous surfaces are superimposed on large cylindrical structures. Several polyhedral structures can be observed, which, due to the effect generated by the vacuum of the analysis chamber, form drops on the analyzed surfaces. No vesicular or polyhedral formations are observable.

## 3. Materials and Methods

### 3.1. Chemicals

The acetone, hexane, methanol, 6-Hydroxy-2,5,7,8-tetramethylchromane-2-carboxylic acid (Trolox), hydrochloric acid, aluminum chloride, ethanol, Folin-Ciocalteu reagent, gallic acid, pectin, 2,2-diphenyl-1-picrylhydrazyl (DPPH), acetonitrile, chlorogenic acid, epicatechin, quercetin, quercetin 3-β-D-glucoside, and kaempferol, formic acid were purchased by Sigma Aldrich (Steinheim am Albuch, Germany). Other reagents, such as sodium bicarbonate, were purchased from Honeywell, Fluka (Seelze, Germany). *Lactobacillus acidophilus* (*Lb. acidophilus*) strain was purchased from Chr. Hansen (Hoersholm, Denmark). de Man, Rogosa and Sharpe agar (MRS agar) were purchased from Merck (Darmstadt, Germany). Pectinex^®^ Ultra Color, consisting of a blend of pectinases and hemicellulases (5000 PGNU/g), was purchased from Novozyme (Novo Holdings, Bagsværd, Denmark). Tyrosinase from mushroom (lyophilized powder, ≥1000 unit/mg solid), 3,4-dihydroxy-L-phenylalanine (DOPA), α-glucosidase from *Saccharomyces cerevisiae* (type I, lyophilized powder, ≥10 units/mg protein), *p*-nitrophenyl-α-D-glucopyranoside), sodium phosphate, pancreatin lipase (111.5 units/mg protein), p-nitrophenyl palmitate, Arabic gum, Triton X-100, kojic acid, Orlistat, and acarbose were purchased from Sigma Aldrich (Steinheim am Albuch, Germany). All reagents and solvents were of analytical and HPLC grade.

### 3.2. Fruits Processing

Fresh rosehips fruit were purchased from a local producer (Braila County, Romania) and were initially sorted based on their uniform color, shape, and size. After sorting, the fruits were washed and processed immediately by blanching, using hot distilled water (70 °C), in a ratio of 1:1 for 15 min. The fruits were divided into three batches, which were processed separately, respecting the same conditions. The fruits were further homogenized using a blender for 3 min at 1000 rpm (Philips HR2100/40 blender, European Community), whereas the resulting pulp was deseeded using a sieve with appropriate dimensions. The resulting pulp was used for an initial phytochemical analysis and further processed. 

The resulting pulp was divided into two equal parts, designed for jellified products and juices manufacturing, respectively. The part designed for juices was enzymatically treated as described in Section 2.5. Both samples were extracted for bioactive compounds using a combined solvent-ultrasound-assisted extraction method. An amount of 1 g from both samples was mixed with 10 mL of methanol-water (90:10, *v/v*) solution, with a solid-liquid ratio of 1:10 (*w/v*). The mixtures were subjected to ultrasound extraction at 35 °C for 2 h. Subsequently, the mixtures were centrifuged at 6000× *g* for 10 min at 4 °C. The supernatant was collected, and the extraction was repeated twice. The collected supernatants (coded as R and RE) were further analyzed for polyphenolic content. 

### 3.3. HPLC Analysis

The separation and identification of the bioactive compounds from the rosehip extracts were carried out by the Agilent 1200 HPLC system equipped with an autosampler, thermostated column compartment, and multiwavelength detector (Agilent Technologies, Santa Clara, CA, USA). Briefly, the C18 Synergy Max RP 80 Å column was used with formic acid binary gradient, as described by Dermengiu et al. [26]. The compounds of interest were identified simultaneously at 280 nm and 320 nm. 

The identification of the polyphenolic compounds from the rosehip extracts was made based on the retention times of the unknown peaks and those of standard solutions of bioactive compounds. Thereafter, the identified compounds were quantified by external calibration curves using the peak area, as shown in Table 1. Data acquisition was made by Chemstation software, version B.04.03 (Agilent Technologies, Santa Clara, CA, USA). Results were expressed in mg/100 g DW extract. 

### 3.4. Jellified Products Manufacturing 

Raw materials of jellified foods production were rosehip pulp, pectin, sugar, and citric acid. The jellified products were obtained with different sugar addition, as follow: pulp and sugar in a ratio of 1:1 (coded as variant 1—V1), pulp and sugar in a ratio of 2:1 (coded as variant 2—V2), pulp and steviol in a ratio of 2:1 (coded as variant 3—V3), and pulp without sugar addition (V). The jellified products were obtained as described by Nistor et al. [17]. In brief, about 450 g of rosehip fruits pulp was mixed with the corresponding amount of sugar or steviol, as well as 0.2% apple pectin (Pektin, Nature Cookta, Budapest, Hungary). The mixtures were heated to 96 °C in a non-stick electric pot (Multicooker Philips HD3037/70, Amsterdam, Netherlands) for a certain heating time adapted to obtain the appropriate consistency. In order to establish the consistency of the jellified products, the cold test was applied, allowing us to estimate the extent of the gelation. The time required by the cross-linked polymer formulas to transit from a free-flowing solution to a gel or semi-gel state was recorded experimentally at 5 min after heating when the preliminary jelly structure was almost obtained, whereas cooling allowed to assure the final specific texture of the jellies. The jellified products were packed into glass jars and stored at room temperature (21 ± 2°C). 

### 3.5. Juices Manufacturing 

The raw material of juices production was about 1 kg of rosehip fruits pulp. The pulp was enzymatically treated with Pectinex^®^ Ultra Color (Novozyme) to promote the extraction of biologically active compounds by the addition of 100 µL of enzyme solution followed by the enzymatic reaction at 60 °C for 1 h. After the enzymatic reaction, the pulp was immediately mixed with apple juice in ratios of 1:0 (coded as J), 1:1 (coded as J1), and 1:3 (coded as J2), packed in glass containers, hermetically sealed and pasteurized at 95 °C for 20 min. The samples were then cooled to 20° ± 1 °C and keep it at room temperature until further analysis. 

### 3.6. Inoculation of Rosehip Pulp with Lactobacillus acidophilus

An amount of 100 g of enzymatically treated pulp was pasteurized at 95 °C for 15 min to inactivate the enzyme and cooled to 40 °C. Prior to inoculation, the mixture was enhanced with pectin (1%) and allowed to hydrate, followed by the pH adjustment at 4.5 with 1.0 N NaOH and sterilization with a UV lamp. In order to obtain a powder with probiotic potential, the pulp was inoculated with an initial cell count of 8.17 log CFU/mL of *Lb. acidophilus*, homogenized for 30 min and freeze-dried (CHRIST Alpha 1–4 LD plus, Osterode am Harz Germany) at −42 °C under the pressure of 10 Pa for 48 h. The resulting powder was collected and packed in metalized bags and kept at 4 °C until further analysis.

### 3.7. Global Phytochemical Characterization of Rosehip Products

In order to estimate the global phytochemical content, an amount of 2.0 g of jellified products, 2 mL of juices, and 1 g of powder were extracted with two different solvents in order to allow the extraction of selected bioactive. Therefore, the samples were mixed with 10 mL of 70% ethanol solution acidified with citric acid (9:1, *v*/*v*) for polyphenolic compounds extraction and with 20 mL of *n*-hexane-acetone solution (3:1, *v*/*v*) for carotenoids extraction, respectively. The phytochemicals were extracted using an ultrasound-assisted protocol at 40 °C for 1 h, followed by centrifugation at 6000× *g* for 10 min at 4 °C. The supernatants were collected, and the extraction was repeated twice. The collected supernatants were further analyzed for phytochemicals content and antioxidant activity.

### 3.8. Total Polyphenols (TP) and Total Flavonoids (TF) Analysis

The TP content was assessed based on the Folin-Ciocâlteu method, as described by Dermengiu et al. [26]. In brief, to a volume of 0.2 mL of the resulting extracts (1:10 diluted in case of juices), 15.8 mL of distilled water was added, followed by the addition of 1 mL of Folin-Ciocâlteu reagent and 3 mL of 20% of Na_2_CO_3_. The mixtures were allowed to react for 1 h in the dark, followed by an absorbance reading at λ = 765 nm (Jenway Scientific Instruments, Essex, UK). Based on a gallic acid (GA) calibration curve, the TP content was expressed as GA equivalents (GAE)/g dry weight (mg GAE/g DW).

The TF content was measured using the aluminum chloride method, involving the sequential addition to a volume of 0.25 mL of resulting extracts of 0.075 mL NaNO_2_ (5%), 0.15 mL of 10% AlCl_3_, and 0.5 mL of 1 M NaOH. The absorbances of the mixtures were measured at 510 nm. The corresponding TF content was expressed in mg catechin equivalents (CE) per g of dry powder (mg CE/g DW). 

### 3.9. Carotenoids Content Evaluation

Spectrophotometric methods were employed for total carotenoid, β-carotene, and lycopene contents estimation in the resulting extracts by selecting different wavelengths, such as 470 nm (β-carotene content), 450 nm (total carotenoids content) and 503 nm (lycopene content). The content of carotenoids was calculated according to Equation (1) [27]:

(1) $$\mathrm{Carotenoids}\;(\mathrm{mg}/g)=\frac{AxM_{w}\times D_{f}}{M_{a}\times L}$$

with: *A*—absorbance of the extracts at corresponding wavelength; *M_w_*—molecular weight, *D_f_*—sample dilution rate, *M_a_*—molar absorptivity (2500 L mol^−1^ cm^−1^, 2590 L mol^−1^ cm^−1^, and 3450 L mol^−1^ cm^−1^, respectively), and *L*—cell diameter of the spectrophotometer (1 cm).

### 3.10. Antiradical Scavenging Activity 

The antiradical scavenging activity against DPPH of the extracts, as described by Fathollahi et al. [28]. The scavenging percentage of DPPH was expressed as mMol Trolox/g DW using a calibration curve.

### 3.11. Texture Analysis of the Jellified Products

The instrumental analysis of jellified products was performed with a Brookfield CT3 Texture Analyzer (Brookfield Ametek, Middleboro, MA, USA), assisted by TexturePro CT V1.5 software. Texture Profile Analysis (TPA) method was used to determine the firmness, adhesiveness, cohesiveness, and springiness of the samples. Immediately after obtaining, the jellified products were packed into cylindrical plastic containers (43 mm diameter and 40 mm height). The containers were kept overnight at room temperature in order to achieve the final gel structure. A double penetration test, using a 38.1 mm diameter acrylic cylinder, was applied until the target distance of 5 mm was reached. Penetration speed was 0.5 mm/s; the trigger was a load at 0.067 N, and the load cell was 9.8 N. Four replicates for each sample were made.

### 3.12. CIEL*a*b* Analysis of the Jellified Products

The jellified products were analyzed for the corresponding color coordinates, including L* (illumination, brightness, 0 black, 100 white), a* (positive value red, negative value green,) and b* (positive value yellow, negative value blue). The color parameters were determined using a CR 410 Chroma Meter (Konica Minolta, Tokyo, Japan) colorimeter.

### 3.13. Nutritional Evaluation of the Jellified Products

The standard AOAC [29] official methods were used to evaluate the moisture, ash, fat, salt, carbohydrates, total sugars, and energy in the samples, whereas the Kjeldahl method was used to determine the protein content. Carbohydrates were calculated by subtracting moisture, protein, fat, salt, and ash. 

### 3.14. Sensorial Analysis of the Jellified Products and Juices

Sensory evaluation of jellified products and juices was performed using the scoring method, using different levels of perception for each attribute on a scale ranging from 1 (very low) to 7 (very high). Ten experienced trained panelists aged between 24 and 56 years were selected and subsequently trained on the sensory-relevant characteristics. The samples were displayed in clear, coded glass jars. Before each analysis, the panelists were asked to cleanse their palate with water, both before the first sample and between samples. The panelists evaluated the following list of sensory attributes: appearance, color, sweet taste, flavor, bitter taste, hardness, fragility, gumminess, mouthfeel, creaminess, and general impression for jellified products and appearance, color, sweet taste, bitter taste, flavor, and general impression for juices. 

### 3.15. Viability of Lb. acidophilus

The viable cells count in the inoculated freeze-dried powder was estimated based on the 10-fold serial dilutions in sterile saline solution (0.9 g NaCl %, *w*/*v*) by the pour plate technique, using MRS agar plates at pH 5.7. The cell counts were evaluated after 72 h of anaerobic incubation at 37 °C and were expressed as the number of colony-forming units CFU/g DW [30].

### 3.16. Inhibitory Activity on Metabolic Syndrome Associated Enzymes

In this study, the inoculated powder containing *Lb. acidophilus* was tested for the ability to inhibit the enzymatic activity of two well-known metabolic syndrome-associated enzymes (α-glucosidase and lipase). Additionally, antityrosinase activity was tested. A preliminary step was performed, allowing to obtain a blank sample absorbance (the mixture containing extract and substrate solution without the enzyme replaced by the buffer). The resulting values were recorded and subtracted from the absorbance. 

For the inhibitory effect, about 10 mg of powder was dissolved in PBS (0.1 M at pH 6.9) and 10-fold diluted to get different ranges of powder concentrations. The inhibition effect was calculated based on Equation (2):

(2) $$\mathrm{Inhibitory}\;\mathrm{effect}\;\left(\%\right)=\frac{A_{c}-A_{s}}{A_{c}}\times 100$$

where: *A_c_* = Absorbance of the control, *A_s_* = Absorbance of the sample.

For all metabolic syndrome-associated enzymes, the inhibitory effects are expressed as a mean of three replicates and given as a 50% inhibition concentration (IC50) calculated from the linear regression of inhibitory activities (%) versus powder concentrations.

The enzyme inhibitory activity of the powder was tested using appropriate substrates, and the results were compared with IC50 values estimated for selected drugs used in current medical practices. Therefore, for the tyrosinase inhibitory capacity, a volume of 800 µL tyrosinase (46 U/mL) was mixed with 500 µL of solutions containing different powder concentrations, followed by the addition of 2 mL of PBS buffer (0.1 M, pH = 6.9) and incubated for 5 min at 37 °C. The enzymatic reaction was initiated by the addition of 1 mL of L-DOPA solution (7.5 mMol PBS), followed by incubation at 37 °C for 30 min. The same procedure was applied for kojic acid as the positive control. The absorbances of mixtures were read at 475 nm. 

The method described by Meziant et al. [31] was used to evaluate the antidiabetic potential of the powder by measuring the potential to inhibit the activity of α-glucosidase. In brief, 50 μL of the solutions containing different powder concentrations were mixed with 100 μL of the enzyme solution (0.5 U/mL in PBS 0.1 M, pH 6.9). The mixtures were allowed to interact for 10 min at 37 °C, whereas the enzymatic reaction was initiated by the addition of 50 μL of 5 mM p-NPG (*p*-nitrophenyl-α-D-glucopyranoside) dissolved PBS. The mixtures were incubated for 20 min at 37 °C. Acarbose was used as the positive control. The absorbances were read at 405 nm.

The in vitro anti-obesity potential of the inoculated powder was assessed by inhibitory ability on the pancreatic lipase. A volume of 100 µL of solutions containing different concentrations of powder was mixed with 50 µL of crude porcine pancreatic lipase dissolved in PBS (1 mg/mL), followed by incubation at 37 °C for 20 min. The reaction was initiated by adding 330 µL of 0.1 M PBS (pH 6.9) supplemented with Triton X-100 (0.6%, *w*/*v*), Arabic gum (0.15%, *w*/*v*), and 20 µL of 10 mM *p*-nitrophenyl palmitate. The same procedure was used for orlistat as the positive control. After 25 min of incubation of the mixtures at 37 °C, the absorbances were monitored at 400 nm. 

### 3.17. Powder Structure and Morphology

The powder morphology was observed by scanning electron microscopy (SEM). The powder was fixed with double-sided tape on the sample holder and later gold-sputter coated, followed by structure and morphology analysis using a Quanta FEG 250 SEM (Hillsboro, OR, USA).

### 3.18. Statistical Analyses 

The analyzes were performed in triplicate, and the results were expressed as mean and standard deviation (SD). The data reported for HPLC analysis are average values for duplicate measurements (*n* = 2) followed by standard deviations. The experimental data were statistically analyzed using One-Way analysis of variance (ANOVA) after checking normality and homoscedasticity tests to identify significant differences. For post-hoc analysis, the Tukey technique with a 95% confidence interval was applied, using a *p* value < 0.05 for statistically significant. Minitab 18 software was used to perform the statistical analysis.

## 4. Conclusions

In our study, different technological approaches were customized for an integrated valorization of rosehip fruits into foods and nutraceuticals, with potential health benefits. The rosehip from Romanian spontaneous flora was used to develop and characterize several technological variants, including jellified products, juices, and a freeze-dried powder containing *Lactobacillus acidophilus*. The phytochemical profile of the corresponding rosehip pulps, without and with enzymatic pretreatment, highlighted the presence of epicatechin as the major compound. The phytochemical profile of the rosehip fruit jellified products was highly influenced by the sugar ratio. Therefore, the sugar addition caused a significant decrease in carotenoid content, ash, moisture, and fat, an increase in carbohydrate content, and better textural parameters. The variants without sugar or with steviol additions showed superior characteristics in carotenoids and color, higher moisture content, and significantly lower content in carbohydrates. The blending of the rosehip juices with apple juices led to a different phytochemical profile, in a ratio-depended manner, influencing the antioxidant activity. The acceptability test of the jellified products and juices revealed the preferences of the panelist towards the samples with lower sugar, whereas the overall impression for the juices increased with the increase of the apple juice content. In order to develop a new perspective for rosehip fruit valorization, the pulp was enriched with pectin and inoculated with *Lactobacillus acidophilus*. The freeze-dried powder showed satisfactory viability of the cells. Moreover, significant anti-tyrosinase, antidiabetic and anti-obesity potential of the powder was suggested when compared with the effect of the drugs used in current medical practice.

Therefore, the results of our study may offer new perspectives for the valorization of spontaneous rosehip fruits, revealing three customized prototypes of functional food and ingredients, mainly due to the high content of nutrients and bioactive compounds (carotenoids and polyphenols), which are linked to the prevention of several diseases.

## Figures and Tables

**Table 1:** Flavonoids and polyphenols from rosehip’s extracts.

Bioactive Compound		Concentration, mg/100 g DW			
		Extract		Extract Treated Pectinex^®^ Ultra Color	
	Calibration Equation 280 and 320 nm	280 nm	320 nm	280 nm	320 nm
Gallic acid	Y = 32,492x + 1935.20 (R^2^ = 0.991) Y = 3650,50x + 43.06 (R^2^ = 0.998)	2.84 ± 0.05 ^1 b^	40.32 ± 4.47 ^a^	9.74 ± 0.24 ^a^	48.75 ± 1.16 ^a^
Chlorogenic acid	Y = 37,938x − 473.39 (R^2^ = 0.995) Y = 65,614x + 420.71 (R^2^ = 0.998)	39.65 ± 9.12 ^a^	3.72 ± 0.14 ^a^	23.12 ± 1.67 ^a^	2.39 ± 0.03 ^b^
Epicatechin	Y = 3441.80x + 188.57 (R^2^ = 0.995)	2077.22 ± 45.62 ^a^	733.93 ± 18.25 ^a^	2145.04 ± 14.95 ^a^	658.86 ± 2.72 ^b^
Quercetin	Y = 62,923x − 668.60 (R^2^ = 0.999) Y = 63,432x − 1092.50 (R^2^ = 0.999)	17.74 ± 1.37 ^b^	29.55 ± 9.12 ^a^	40.23 ± 0.48 ^a^	6.24 ± 0.08 ^a^
Kaempferol	Y = 12,672x + 947.22 (R^2^ = 0.992) Y = 51,624x + 2429.90 (R^2^ = 0.991)	0.90 ± 0.06 ^b^	0.27 ± 0.02 ^b^	5.06 ± 0.05 ^a^	0.56 ± 0.01 ^a^
Quercetin 3-β-D-glucoside	Y = 1875.80x − 6.97 (R^2^ = 0.999) Y = 70,182x + 1690 (R^2^ = 0.998)	n.d.	798.54 ± 24.73 ^a^	25.74 ± 0.77 ^a^	n.d.

^1.^ Average values for duplicate measurements (n = 2) ± standard deviations (n = 3); n.d.—not determined. Different letters in one row at the same wavelength denote significant differences (*p* < 0.05) between samples.

**Table 2:** Phytochemical profile and antioxidant activity by DPPH assay of jellified products.

Variants	P	V	V1	V2	V3
Total carotenoids (mg/g DW)	185.17 ± 1.68 ^a^	89.36 ± 0.06 ^b^	9.66 ± 0.26 ^d^	5.38 ± 0.04 ^e^	39.22 ± 1.16 ^c^
β-carotene (mg/g DW)	148.98 ± 1.06 ^a^	83.52 ± 0.10 ^b^	7.95 ± 0.50 ^d^	5.24 ± 0.02 ^e^	38.06 ± 0.10 ^c^
Lycopene (mg/g DW)	81.16 ± 2.42 ^a^	42.33 ± 0.18 ^b^	5.11 ± 0.21 ^d^	2.86 ± 0.01 ^e^	18.47 ± 0.18 ^c^
Antioxidant activity (mMol Trolox/g DW)	36.44 ± 2.45 ^a^	8.19 ± 0.80 ^c^	6.24 ± 0.16 ^c^	6.05 ± 0.29 ^c^	20.16 ± 0.31 ^b^

P—rosehip pulp, V—jellified product without sugar addition, V1—jellified pulp with sugar addition (ratio of 1:1), V2—jellified product from pulp with sugar addition (ratio of 2:1), V3—jellified product from pulp with steviol addition (ratio of 2:1). Values are represented as mean ± standard deviations (n = 5). Superscript values in the same row showing distinct letters (a, b, c, d, e) are significantly different at *p* < 0.05, based on the Tukey test.

**Table 3:** Textural parameters of rosehip jellified products.

Variants	Firmness, N	Adhesiveness, mJ	Cohesiveness, -	Springiness, mm
V	1.14 ± 0.03 ^a^	0.22 ± 0.02 ^b^	0.41 ± 0.01 ^b^	4.76 ± 0.11 ^b^
V1	3.62 ± 0.08 ^b^	0.76 ± 0.03 ^a^	0.68 ± 0.02 ^b^	3.87 ± 0.23 ^b^
V2	2.85 ± 0.03 ^b^	0.54 ± 0.02 ^b^	0.65 ± 0.01 ^b^	4.12 ± 0.12 ^b^
V3	1.61 ± 0.02 ^a^	0.31 ± 0.03 ^b^	0.46 ± 0.01 ^b^	4.79 ± 0.15 ^b^

V—jellified product without sugar addition, V1—jellified pulp with sugar addition (ratio of 1:1), V2—jellified product from pulp with sugar addition (ratio of 2:1), V3—jellified product from pulp with steviol addition (ratio of 2:1). Values are represented as mean ± standard deviations (n = 5). Superscript values in the same columns showing distinct letters (a, b) are significantly different at *p* < 0.05, based on the Tukey test.

**Table 4:** Color parameters of rosehip jellified products.

Variants	L*	a*	b*	C	h
M	32.10 ± 0.5 ^b^	6.39 ± 0.1 ^b^	20.94 ± 0.5 ^b^	38.28 ± 0.3 ^b^	33.25 ± 0.5 ^a^
V	22.39 ± 1.0 ^b^	9.89 ± 0.2 ^b^	28.44 ± 0.2 ^b^	27.72 ± 0.1 ^b^	66.65 ± 1.0 ^a^
V1	20 ± 2.0 ^b^	4.86 ± 1.0 ^b^	21.59 ± 0.7 ^b^	23.12 ± 0.5 ^b^	77.79 ± 1.0 ^a^
V2	18.86 ± 2.0 ^b^	7.02 ± 0.2 ^b^	21.24 ± 0.4 ^b^	22.40 ± 0.3 ^b^	71.35 ± 0.5 ^a^
V3	29.61 ± 0.7 ^b^	7.01 ± 0.3 ^b^	17.86 ± 1.0 ^b^	19.18 ± 0.1 ^b^	22.47 ± 0.3 ^a^

M—unprocessed rosehip pulp, V—jellified product without sugar addition, V1—jellified pulp with sugar addition (ratio of 1:1), V2—jellified product from pulp with sugar addition (ratio of 2:1), V3—jellified product from pulp with steviol addition (ratio of 2:1). L* (lightness/darkness), a* (red/green) and b* (yellow/blue), h*—hue angle, C*—chroma, color intensity. Values are represented as mean ± standard deviations (n = 5). Superscript values in the same columns showing distinct letters (a, b) are significantly different at *p* < 0.05, based on the Tukey test.

**Table 5:** The proximate analysis and caloric values of the jellified samples.

Characteristics (%)	V	V1	V2	V3
Salt, of which sodium	0.06 ± 0.001 ^b^ 0.024 ± 0.001 ^b^	0.08 ± 0.001 ^a^ 0.032 ± 0.001 ^a^	0.08 ± 0.007 ^a^ 0.032 ± 0.001 ^a^	0.06 ± 0.002 ^b^ 0.024 ± 0.002 ^b^
Ash	0.951 ± 0.001 ^a^	0.355 ± 0.001 ^d^	0.473 ± 0.001 ^c^	0.803 ± 0.003 ^b^
Moisture	78.96 ± 0.79 ^a^	5.21 ± 0.19 ^d^	33.4 ± 1.52 ^c^	66.92 ± 2.17 ^b^
Fat	0.12 ± 0.01 ^a^	0.07 ± 0.01 ^b^	0.04 ± 0.001 ^c^	0.12 ± 0.01 ^a^
Proteins	0.20 ± 0.01 ^a^	0.20 ± 0.01 ^a^	0.20 ± 0.01 ^a^	0.20 ± 0.01 ^a^
Carbohydrate, of which sugar	19.9 ± 0,10 ^d^ 3.69 ± 0.02 ^d^	93.9 ± 0.21 ^a^ 46.48 ± 0.02 ^a^	65.7 ± 0.12 ^b^ 21.95 ± 0.01 ^b^	32.00 ± 0.01 ^c^ 5.44 ± 0.03 ^c^
Energy value (kcal) (kj)	81.48 ± 0.05 ^d^ 346.14 ± 2.36 ^d^	337.8 ± 1.23 ^a^ 1605.7 ± 11.05 ^a^	264.7 ± 5.62 ^b^ 1125.2 ± 5.46 ^b^	129.9 ± 2.84 ^c^ 551.8 ± 0.98 ^c^

V—jellified product without sugar addition, V1—jellified pulp with sugar addition (ratio of 1:1), V2—jellified product from pulp with sugar addition (ratio of 2:1), V3—jellified product from pulp with steviol addition (ratio of 2:1). Values are represented as mean ± standard deviations (n = 5). Superscript values in the same row showing distinct letters (a, b, c, d) are significantly different at *p* < 0.05, based on the Tukey test.

**Table 6:** Phytochemical profile and antioxidant activity by DPPH assay of Rosehip juices.

Variants	P	J	J1	J2
Total carotenoid (mg/100 mL)	144.06 ± 0.33 ^a^	141.74 ± 1.16 ^b^	59.92 ± 0.17 ^c^	34.50 ± 1.22 ^d^
β-carotene (mg/100 mL)	134.61 ± 0.20 ^b^	140.70 ± 0.22 ^a^	57.68 ± 0.33 ^c^	32.08 ± 1.12 ^d^
Lycopene (mg/100 mL)	79.13 ± 0.41 ^a^	63.97 ± 0.53 ^b^	31.66 ± 0.12 ^c^	17.14 ± 0.72 ^d^
Total polyphenolic content (mg GAE/100 mL)	58.91 ± 2.89 ^a^	57.67 ± 3.83 ^a^	126.26 ± 2.38 ^b^	207.49 ± 1.04 ^c^
Antioxidant activity (mMol trolox/100 mL)	27.45 ± 1.68 ^a^	5.77 ± 0.38 ^d^	12.63 ± 0.64 ^c^	20.75 ± 1.40 ^b^

P—Rosehip pulp after enzymatic treatment; J—juice from rosehip pulp; J1—juice from rosehip pulp with addition of apple juice (ratio 1:1, J2—juice from rosehip pulp with addition of apple juice (ratio 1:3). Values are represented as mean ± standard deviations (n = 5). Superscript values in the same row with distinct letters (a, b, c, d) are significantly different at *p* < 0.05, based on the Tukey test.

**Table 7:** Sensorial analysis of jellified products.

Samples/Attributes	V	V1	V2	V3
Appearance	5.4 ± 0.84 ^bc^	4.5 ± 0.84 ^c^	6.4 ± 0.69 ^a^	6.1 ± 0.73 ^ab^
Color	5.4 ± 1.20 ^a^	5.5 ± 0.52 ^a^	6 ± 0.47 ^a^	6.3 ± 0.82 ^a^
Sweet taste	1.7 ± 0.67 ^b^	6.3 ± 1.05 ^a^	6.2 ± 1.03 ^a^	5.8 ± 0.78 ^a^
Bitter taste	1.7 ± 0.67 ^a^	1.4 ± 0.51 ^a^	1.3 ± 0.48 ^a^	1.6 ± 0.51 ^a^
Flavor	4.2 ± 1.22 ^b^	4.7 ± 0.67 ^b^	6 ± 0.47 ^a^	5.00 ± 1.05 ^ab^
Hardness	2.1 ± 0.56 ^bc^	6.9 ± 0.31 ^a^	2.5 ± 0.70 ^b^	1.7 ± 0.67 ^c^
Fragility	3.2 ± 0.63 ^b^	5.4 ± 0.69 ^a^	2.6 ± 0.84 ^b^	3.1 ± 0.73 ^b^
Gumminess	1.6 ± 0.69 ^c^	5.9 ± 0.73 ^a^	3.00 ± 0.66 ^b^	1.5 ± 0.70 ^c^
Mouthfeel	1.8 ± 0.78 ^b^	6.6 ± 0.78 ^a^	1.8 ± 0.78 ^b^	1.9 ± 0.73 ^b^
Creaminess	4.5 ± 1.35 ^a^	2.4 ± 1.05 ^b^	5.3 ± 1.05 ^a^	5.7 ± 0.94 ^a^
Overall impression	5.1 ± 1.19 ^a^	3.5 ± 0.78 ^b^	6.2 ± 0.78 ^a^	5.7 ± 1.25 ^a^

V—jellified product without sugar addition, V1—jellified pulp with sugar addition (ratio of 1:1), V2—jellified product from pulp with sugar addition (ratio of 2:1), V3—jellified product from pulp with steviol addition (ratio of 2:1). Values are represented as mean ± standard deviations (n = 7). Superscript values in the same row with distinct letters (a, b, c) are significantly different at *p* < 0.05, based on the Tukey test.

**Table 8:** Sensorial analysis of juices.

Samples/Attributes	J	J1	J2
Appearance	6.4 ± 1.07 ^a^	6.1 ± 0.87 ^a^	5.3 ± 1.05 ^a^
Color	6.4 ± 0.96 ^a^	6.3 ± 0.67 ^a^	5.6 ± 1.42 ^a^
Sweet taste	2 ± 1.49 ^b^	3.9 ± 1.52 ^a^	4.9 ± 1.37 ^a^
Bitter taste	2.4 ± 1.89 ^a^	1.3 ± 0.67 ^a^	1.6 ± 0.69 ^a^
Flavor	4.8 ± 1.75 ^a^	5.1 ± 1.37 ^a^	5.6 ± 1.17 ^a^
Overall impression	4.8 ± 1.75 ^a^	5.6 ± 1.07 ^a^	5.9 ± 1.10 ^a^

P—Rosehip pulp after enzymatic treatment; J—juice from rosehip pulp; J1—juice from rosehip pulp with addition of apple juice (ratio 1:1, J2—juice from rosehip pulp with addition of apple juice (ratio 1:3). Values are represented as mean ± standard deviations (n = 5). Superscript values in the same row with distinct letters (a, b) are significantly different at *p* < 0.05, based on the Tukey method.

**Table 9:** The inhibitory activity (IC50, mg/mL) of freeze-dried *Lactobacillus acidophilus* inoculated powder based on rosehip fruits pulp.

Enzyme	α-Glucosidase	Lipase	Tyrosinase
Powder	2.92 ± 0.83 *	1.21 ± 0.08	0.23 ± 0.05
Acarbose	3.97 ± 0.62	-	-
Orlistat	-	1.23 ± 0.09	-
Kojic acid	-	-	1.12 ± 0.14

* Mean ± standard deviations of three replicates.

## Data Availability

Not applicable.

## References

[1] Ozdemir N., Pashazadeh H., Zannou O., Koca I. (2022). Phytochemical content, and antioxidant activity, and volatile compounds associated with the aromatic property, of the vinegar produced from rosehip fruit (*Rosa canina* L.). LWT.

[2] Elmastaş M., Demir A., Genç N., Dölek U., Güneş M. (2017). Changes in Flavonoid and Phenolic Acid Contents in Some Rosa species During Ripening. Food Chem..

[3] Patel S. (2017). Rose hip as an underutilized functional food: Evidence-based review. Trends Food Sci. Technol..

[4] Petkova N., Ognyanov M., Kirchev M., Stancheva M. (2021). Bioactive compounds in water extracts prepared from rosehip-containing herbal blends. J. Food Process. Preserv..

[5] Winther K., Vinther Hansen A.S., Campbell-Tofte J. (2016). Bioactive ingredients of rose hips (*Rosa canina* L.) with special reference to antioxidative and anti inflammatory properties: In vitro studies. Botanics.

[6] Bruneau A., Starr J.R., Joly S. (2007). Phylogenetic relationships in the genus Rosa: New evidence from chloroplast DNA sequences and an appraisal of current knowledge. Syst. Bot..

[7] Ghazghazi H., Miguel M.G., Hasnaoui B., Sebei H., Ksontini M., Figueiredo A.C., Pedro L.G., Barroso J.G. (2010). Phenols, essential oils, and carotenoids of *Rosa canina* from Tunisia and their antioxidant activities. Afr. J. Biotechnol..

[8] Goztepe B., Kayacan S., Bozkurt F., Tomas M., Sagdic O., Karasu S. (2022). Drying kinetics, total bioactive compounds, antioxidant activity, phenolic profile, lycopene and β-carotene content and color quality of Rosehip dehydrated by different methods. LWT.

[9] Medveckienė B., Kulaitienė J., Jarienė E., Vaitkevičienė N., Hallman E. (2020). Carotenoids, Polyphenols, and Ascorbic Acid in Organic Rosehips (*Rosa* spp.) Cultivated in Lithuania. Appl. Sci..

[10] Ilyasoğlu H. (2014). Characterization of Rosehip (*Rosa canina* L.) Seed and Seed Oil. Int. J. Food Prop..

[11] Yildiz O., Alpaslan M. (2012). Properties of Rose Hip Marmalades. Food Technol. Biotechnol..

[12] Ercisli S. (2007). Chemical composition of fruits in some rose (*Rosa* spp.) species. Food Chem..

[13] Angelov G., Boyadzhieva S., Georgieva S. (2014). Rosehip extraction; process optimization and antioxidant capacity of extracts. Central Eur. J. Chem..

[14] Medveckienė B., Kulaitienė J., Levickienė D., Hallmann E. (2021). The effect of ripening stages on the accumulation of carotenoids, polyphenols and vitamin C in rosehip species/cultivars. Appl. Sci..

[15] Figueroa L.E., Genovese D.B. (2019). Fruit jellies enriched with dietary fibre: Development and characterization of a novel functional food product. LWT.

[16] Ben Rejeb I., Dhen N., Kassebi S., Gargouri M. (2020). Quality evaluation and functional properties of reduced sugar jellies formulated from citrus fruits. J. Chem..

[17] Nistor O.-V., Bolea C.A., Andronoiu D.G., Cotârleț M., Stănciuc N. (2021). Attempts for Developing Novel Sugar-Based and Sugar-Free Sea Buckthorn Marmalades. Molecules.

[18] Bourne M.C. (2002). Principles of objective texture measurement. Food Texture and Viscosity. Concept and Measurement.

[19] Featherstone S. (2016). Jams, jellies, and related products. A Complete Course in Canning and Related Processes, Volume 3: Processing Procedures for Canned Food Products.

[20] Igual M., Chi M.S., Paucean A., Vodnar D.C., Muste S., Man S., Martínez-Monzó J., García-Segovia P. (2021). Valorization of Rose Hip (*Rosa canina*) Puree Co-Product in Enriched Corn Extrudates. Foods.

[21] Drożdż W., Tomaszewska-Ciosk E., Zdybel E., Boruczkowska H., Boruczkowski T., Regiec P. (2014). Effect of apple and rosehip pomaces on colour, total phenolics and antioxidant activity of corn extruded snacks. Pol. J. Chem. Technol..

[22] Ramadan M.F., Kuddus M. (2019). Enzymes in Fruit Juice Processing. Enzymes in Food Biotechnology Production, Applications, and Future Prospects.

[23] Pashazadeh O., Özdemir N., Zannou O., Koca I., Mayıs O. (2021). Antioxidant capacity, phytochemical compounds, and volatile compounds related to aromatic property of vinegar produced from black rosehip (*Rosa pimpinellifolia* L.) juice. Food Biosci..

[24] Turgut T., Çetin B., Erdoğan A., Gürses M. (2005). Some microbiological characteristics of rosehip yoghurt inoculated with *Lactobacillus acidophilus* DSMZ 20079. Acta Hortic..

[25] Milea Ș.A., Vasile M.A., Crăciunescu O., Prelipcean A.-M., Bahrim G.E., Râpeanu G., Stănciuc N. (2020). Co-Microencapsulation of flavonoids from yellow onion skins and lactic acid bacteria lead to multifunctional ingredient for nutraceutical and pharmaceutics applications. Pharmaceutics.

[26] Dermengiu N.E., Milea Ș.A., Păcularu Burada B., Stanciu S., Cîrciumaru A., Râpeanu G., Stănciuc N. (2022). A Dark Purple Multifunctional Ingredient from Blueberries Pomace enhanced with Lactic acid bacteria for Various Applications. J. Food Sci..

[27] Souza A.L.R., Hidalgo-Chávez D.W., Pontes S.M., Gomes F.S., Cabral L.M.C., Tonon L.V. (2018). Microencapsulation by spray drying of a lycopene-rich tomato concentrate: Characterization and stability. LWT.

[28] Fathollahi M., Aminzare M., Mohseni M., Hassanzadazar H. (2019). Antioxidant capacity, antimicrobial activities and chemical composition of *Pistacia atlantica* subsp. kurdica essential oil. Vet. Res. Forum.

[29] AOAC (1990). Official Methods of Analysis 15th Edition of the Association of Official Analytical Chemists.

[30] Vasile M.A., Milea Ș.-A., Enachi E., Barbu V., Cîrciumaru A., Bahrim G.E., Râpeanu G., Stănciuc N. (2020). Functional enhancement of bioactives from black beans and lactic acid bacteria into an innovative food ingredient by co-microencapsulation. Food Bioproc. Technol..

[31] Meziant L., Bachir-bey M., Bensouici C., Saci F., Boutiche M., Louaileche H. (2021). Assessment of inhibitory properties of flavonoid-rich fig (*Ficus carica* L.) peel extracts against tyrosinase, α-glucosidase, urease and cholinesterases enzymes, and relationship with antioxidant activity. Eur. J. Integr. Med..

